# Variance components and correlations of female fertility traits in Chinese Holstein population

**DOI:** 10.1186/s40104-017-0189-x

**Published:** 2017-07-01

**Authors:** Aoxing Liu, Mogens Sandø Lund, Yachun Wang, Gang Guo, Ganghui Dong, Per Madsen, Guosheng Su

**Affiliations:** 1 Laboratory of Animal Genetics, Breeding and Reproduction, Ministry of Agriculture of China, National Engineering Laboratory of Animal Breeding, College of Animal Science and Technology, China Agricultural University, Beijing, 100193 China; 2 Center for Quantitative Genetics and Genomics, Department of Molecular Biology and Genetics, Aarhus University, 8830 Tjele, Denmark; 3 Beijing Sunlon Livestock Development Co., Ltd, Beijing, 100176 China

**Keywords:** Chinese Holsteins, Female fertility, Genetic correlation, Heritability

## Abstract

**Background:**

The objective of the present study was to estimate (co)variance components of female fertility traits in Chinese Holsteins, considering fertility traits in different parities as different traits. Data on 88,647 females with 215,632 records (parities) were collected during 2000 to 2014 from 32 herds in the Sanyuan Lvhe Dairy Cattle Center, Beijing, China. The analyzed female fertility traits included interval from calving to first insemination, interval from first to last insemination, days open, conception rate at first insemination, number of inseminations per conception and non-return rates within 56 days after first insemination.

**Results:**

The descriptive statistics showed that the average fertility of heifers was superior to that of cows. Moreover, the genetic correlations between the performances of a trait in heifers and in cows were all moderate to high but far from one, which suggested that the performances of a trait in heifers and cows should be considered as different but genetically correlated traits in genetic evaluations. On the other hand, genetic correlations between performances of a trait in different parities of cows were greater than 0.87, with only a few exceptions, but variances were not homogeneous across parities for some traits. The estimated heritabilities of female fertility traits were low; all were below 0.049 (except for interval from calving to first insemination). Additionally, the heritabilities of the heifer interval traits were lower than those of the corresponding cow interval traits. Moreover, the heritabilities of the interval traits were higher than those of the threshold traits when measuring similar fertility functions. In general, estimated genetic correlations between traits were highly consistent with the biological categories of the female fertility traits.

**Conclusions:**

Interval from calving to first insemination, interval from first to last insemination and non-return rates within 56 days after first insemination are recommended to be included in the selection index of the Chinese Holstein population. The parameters estimated in the present study will facilitate the development of a genetic evaluation system for female fertility traits to improve the reproduction efficiency of Chinese Holsteins.

## Background

Female fertility traits are the most economically functional traits affecting the efficiency of the dairy industry. Cows with excellent fertility resume cycles soon after calving and become pregnant when properly inseminated [1]. According to previous studies, poor reproduction has become a worldwide problem and the most probable cause for culling [2, 3]. Due to the unfavorable correlations between yield and female fertility traits, intensive selection for milk production over the past five decades has led to a strong decline in cow reproduction, particularly in high-producing populations [4, 5]. Moreover, the heritabilities of most fertility traits are typically below 5% [6–8], thus, selection for female fertility is not as efficient as selection for production traits. Therefore, the reproductive decline in modern dairy cattle cannot be ignored, as the costs of extra inseminations, fertility treatments and involuntary culling are increasing.

Although the heritabilities of reproduction traits are relatively low, their additive genetic variances have remained sufficiently high to perform effective selection. Nordic countries (including Denmark, Finland and Sweden) are pioneers in applying fertility traits in dairy cattle breeding and have implemented fertility traits in their selection index since 1994. The Nordic Cattle Genetic Evaluation has shown that the genetic trend for the female fertility index of Holstein bulls has increased by approximately 1% per year since 2000 (http://www.sweebv.info/ba52nycknav.aspx). Furthermore, the results of routine genetic evaluations in Canada (https://www.cdn.ca/) and the United States (http://www.holsteinusa.com/) have shown that the decline in the genetic trend of fertility traits has flattened and even slightly reversed after increasing the weight of fertility traits in the selection index [9]. In a recent test run of the multiple across-country evaluation (MACE) by Interbull in August 2016, 21 countries participated in the genetic evaluation of female fertility traits [10]. In China, however, an insemination and calving record collection system has not yet been implemented on the national scale, therefore, female fertility traits are still not included in the current selection index for Chinese Holsteins. Preliminary results from a genetic evaluation of five fertility traits (age at first insemination, number of inseminations per conception, days from calving to first insemination, days open and calving interval) in Chinese Holsteins have been recently published [11]. However, that dataset was relatively small and did not include several internationally recommended female fertility traits, such as conception rate and non-return rate at first insemination. Moreover, the main limitation of the previous analysis is that the performances of a trait in heifers and cows were simply treated as the same trait, which differs from various previous genetic evaluation studies [6, 12]. The physiological status of cows changes considerably after first calving [13], thus, the performances of heifers and cows have typically been considered biologically distinct [14, 15]. Nevertheless, the genetic relationships among female fertility traits across multiple parities in dairy cattle remain less well investigated.

With respect to the models and algorithms typically used in the genetic evaluation of female fertility traits, many studies have shown that linear models perform similarly to threshold models but with less computational demand, consequently, linear models have been widely used in routine genetic evaluations [12, 16]. In addition, animal and sire models are currently the most frequently implemented models in routine genetic evaluations. Compared with sire models, animal models are theoretically superior for estimating variance components and breeding values. Several previous studies have reported that animal models provide more accurate predictions than sire models [17–19].

The aim of the present study was to assess the genetic relationships across multiple parities, estimate the variance and covariance components, and calculate the heritabilities and genetic correlations of female fertility traits in Chinese Holsteins. The results of this study will facilitate the development of a routine genetic evaluation system for female fertility traits, resulting in a more balanced dairy cattle breeding program in China.

## Methods

### Data

Field records of insemination and calving from 2000 to 2014 were collected from 32 herds in the Sanyuan Lvhe Dairy Cattle Center, Beijing, China. The following traits were recorded: interval from calving to first insemination (ICF), days open (DO), interval from first to last insemination (IFL), conception rate at first insemination (CR), number of inseminations per conception (AIS) and non-return rate within 56 days after first insemination (NRR56). For binary traits, CR was coded as 0 (failure) or 1 (success), and NRR56 was coded as 1 when there was no subsequent insemination within 56 days after the first insemination and 0 otherwise. All traits recorded before first calving were considered as heifer traits and were coded as parity 0. Traits measured in first-lactation cows were coded as parity 1, and the same strategy was used for the remaining lactations. Among these traits, ICF and DO were available only for cows. All the remaining traits were measured for both heifers and cows. In this context, h and c were used as suffixes in trait abbreviations to distinguish heifers and cows.

Most fertility traits included censored records, as mistakes in recording and some culling events arose during data collection or fertility events were still in progress. The last insemination with a positive pregnancy test result or with the following calving record was considered a verified conception. In the present data, 5.92% of heifers and 13.92% of cow-lactations showed no verified conception from the last insemination. In such cases, the IFL and AIS were calculated according to the last insemination, and the expectation of the trait was added as a penalty. Based on uncensored data, the expectations of IFLh, IFLc, AISh and AISc were 21 days, 43 days, 1.5, and 2.0, respectively. Similarly, for cows without ascertained conception, the penalty for DO was the same as that for IFL. To further reduce the proportion of censored records, we only used data for females with birth years from 1999 to 2013, insemination years from 2000 to 2014, and calving years from 2000 to 2014. To reduce possible selection bias, cows without heifer records were also removed from the data. Moreover, the data in the analysis contained only females inseminated by AI bulls. Other criteria for data editing included age at first insemination between 270 and 900 days, age at first calving between 500 and 1100 days, gestation length between 240 and 300 days, ICF between 20 and 230 days, IFL less than 365 days and AIS less than eight times per parity. Records with values smaller than the lower limit were deleted as outliers, while those with values larger than the upper limit were set as the upper limit.

Based on the distribution of records across parities for each trait, only data from heifers and the first three lactations were used in the analysis. After editing, the total number of cows in the final dataset was 88,647, and the total number of records (parities) was 215,632. The numbers of observations available for heifers and the first three parities of cows were 88,647, 60,022, 41,858 and 25,105, respectively. Heifer records comprised 41.11% of the entire dataset.

Full pedigree data were provided by the Dairy Association of China (Beijing, China), and each animal was traced back as many generations as possible. Unknown parents were assigned into 16 phantom groups, based on sex and birth year. In total, the pedigree included 179,939 females and 4310 males, with birth years from 1930 to 2013. Considering the number of heifers/cows in the present study, the size of pedigree was small. This was determined by the data structure. In the current study, the phenotypic data covered a long period, and the females came from 32 herds during birth year 1999 to 2013. Thus, the phenotypic data already covered a large number of female ancestors (such like mother, grandmother and so on). Therefore, the number of animals in the pedigree was only about two times as large as the number of animals with fertility records, even though the heifers/cows were traced back as many generations as possible.

### Models

Five linear Gaussian animal models were used to estimate the (co)variance components of female fertility traits. First, data across multiple parities were analyzed using a multi-trait model in which the performances of a trait in different parities were treated as different traits. For each of the traits defined both in heifers and cows (IFL, CR, AIS and NRR56), a four-trait model was used.

1 $$ \left[\begin{array}{l}{\mathbf{y}}_{\mathbf{0}}\hfill \\ {}{\mathbf{y}}_{\mathbf{1}}\hfill \\ {}{\mathbf{y}}_{\mathbf{2}}\hfill \\ {}{\mathbf{y}}_{\mathbf{3}}\hfill \end{array}\right]=\left[\begin{array}{llll}{\mathbf{X}}_{\mathbf{0}}\hfill & \mathbf{0}\hfill & \mathbf{0}\hfill & \mathbf{0}\hfill \\ {}\mathbf{0}\hfill & {\mathbf{X}}_{\mathbf{1}}\hfill & \mathbf{0}\hfill & \mathbf{0}\hfill \\ {}\mathbf{0}\hfill & \mathbf{0}\hfill & {\mathbf{X}}_{\mathbf{2}}\hfill & \mathbf{0}\hfill \\ {}\mathbf{0}\hfill & \mathbf{0}\hfill & \mathbf{0}\hfill & {\mathbf{X}}_{\mathbf{3}}\hfill \end{array}\right]\left[\begin{array}{l}{\beta}_{\mathbf{0}}\hfill \\ {}{\beta}_{\mathbf{1}}\hfill \\ {}{\beta}_{\mathbf{2}}\hfill \\ {}{\beta}_{\mathbf{3}}\hfill \end{array}\right]+\left[\begin{array}{llll}{Z}_{\mathbf{0}}\hfill & \mathbf{0}\hfill & \mathbf{0}\hfill & \mathbf{0}\hfill \\ {}\mathbf{0}\hfill & {Z}_{\mathbf{1}}\hfill & \mathbf{0}\hfill & \mathbf{0}\hfill \\ {}\mathbf{0}\hfill & \mathbf{0}\hfill & {Z}_{\mathbf{2}}\hfill & \mathbf{0}\hfill \\ {}\mathbf{0}\hfill & \mathbf{0}\hfill & \mathbf{0}\hfill & {Z}_{\mathbf{3}}\hfill \end{array}\right]\left[\begin{array}{l}{a}_{\mathbf{0}}\hfill \\ {}{a}_{\mathbf{1}}\hfill \\ {}{a}_{\mathbf{2}}\hfill \\ {}{a}_{\mathbf{3}}\hfill \end{array}\right]+\left[\begin{array}{l}{\mathbf{e}}_{\mathbf{0}}\hfill \\ {}{\mathbf{e}}_{\mathbf{1}}\hfill \\ {}{\mathbf{e}}_{\mathbf{2}}\hfill \\ {}{\mathbf{e}}_{\mathbf{3}}\hfill \end{array}\right] $$

For traits only defined in cows (ICF and DO), a three-trait model was used.

2 $$ \left[\begin{array}{l}{\mathbf{y}}_{\mathbf{1}}\hfill \\ {}{\mathbf{y}}_{\mathbf{2}}\hfill \\ {}{\mathbf{y}}_{\mathbf{3}}\hfill \end{array}\right]=\left[\begin{array}{lll}{\mathbf{X}}_{\mathbf{1}}\hfill & \mathbf{0}\hfill & \mathbf{0}\hfill \\ {}\mathbf{0}\hfill & {\mathbf{X}}_{\mathbf{2}}\hfill & \mathbf{0}\hfill \\ {}\mathbf{0}\hfill & \mathbf{0}\hfill & {\mathbf{X}}_{\mathbf{3}}\hfill \end{array}\right]\left[\begin{array}{l}{\beta}_{\mathbf{1}}\hfill \\ {}{\beta}_{\mathbf{2}}\hfill \\ {}{\beta}_{\mathbf{3}}\hfill \end{array}\right]+\left[\begin{array}{lll}{Z}_{\mathbf{1}}\hfill & \mathbf{0}\hfill & \mathbf{0}\hfill \\ {}\mathbf{0}\hfill & {Z}_{\mathbf{2}}\hfill & \mathbf{0}\hfill \\ {}\mathbf{0}\hfill & \mathbf{0}\hfill & {Z}_{\mathbf{3}}\hfill \end{array}\right]\left[\begin{array}{l}{a}_{\mathbf{1}}\hfill \\ {}{a}_{\mathbf{2}}\hfill \\ {}{a}_{\mathbf{3}}\hfill \end{array}\right]+\left[\begin{array}{l}{\mathbf{e}}_{\mathbf{1}}\hfill \\ {}{\mathbf{e}}_{\mathbf{2}}\hfill \\ {}{\mathbf{e}}_{\mathbf{3}}\hfill \end{array}\right] $$

In addition, cow traits (ICF, DO, IFLc, CRc, AISc and NRR56c) were further analyzed by pooling data over parities and using a single-trait model treating records in multiple parities as repeated measurements.

3 $$ \boldsymbol{y}=\boldsymbol{X}\boldsymbol{\beta } +\boldsymbol{Za}+\boldsymbol{Wpe}+\boldsymbol{e} $$

Correlations between a pair of heifer traits and a pair of cow traits were estimated using a two-trait model. For a pair of heifer traits (IFLh, CRh, AISh and NRR56h), the following two-trait model was used:

4 $$ \left[\begin{array}{l}{\mathbf{y}}_1\hfill \\ {}{\mathbf{y}}_2\hfill \end{array}\right]=\left[\begin{array}{ll}{\mathbf{X}}_1\hfill & \mathbf{0}\hfill \\ {}\mathbf{0}\hfill & {\mathbf{X}}_2\hfill \end{array}\right]\left[\begin{array}{l}{\beta}_1\hfill \\ {}{\beta}_2\hfill \end{array}\right]+\left[\begin{array}{ll}{Z}_1\hfill & \mathbf{0}\hfill \\ {}\mathbf{0}\hfill & {Z}_2\hfill \end{array}\right]\left[\begin{array}{l}{a}_1\hfill \\ {}{a}_2\hfill \end{array}\right]+\left[\begin{array}{l}{\mathbf{e}}_1\hfill \\ {}{\mathbf{e}}_2\hfill \end{array}\right] $$

For a pair of cow traits (ICF, DO, IFLc, CRc, AISc and NRR56c), the following two-trait model was used:

5 $$ \left[\begin{array}{c}\hfill {\mathbf{y}}_1\hfill \\ {}\hfill {\mathbf{y}}_2\hfill \end{array}\right]=\left[\begin{array}{ll}{\mathbf{X}}_1\hfill & \mathbf{0}\hfill \\ {}\mathbf{0}\hfill & {\mathbf{X}}_2\hfill \end{array}\right]\left[\begin{array}{c}\hfill {\beta}_{\mathbf{0}}\hfill \\ {}\hfill {\beta}_{\mathbf{1}}\hfill \end{array}\right]+\left[\begin{array}{ll}{Z}_1\hfill & \mathbf{0}\hfill \\ {}\mathbf{0}\hfill & {Z}_2\hfill \end{array}\right]\left[\begin{array}{c}\hfill {a}_1\hfill \\ {}\hfill {a}_2\hfill \end{array}\right]+\left[\begin{array}{ll}{W}_1\hfill & \mathbf{0}\hfill \\ {}\mathbf{0}\hfill & {W}_2\hfill \end{array}\right]\left[\begin{array}{c}\hfill {pe}_1\hfill \\ {}\hfill {pe}_2\hfill \end{array}\right]+\left[\begin{array}{c}\hfill {\mathbf{e}}_1\hfill \\ {}\hfill {\mathbf{e}}_2\hfill \end{array}\right] $$

In the above models, ***y***_***i***_ was the vector of observations (*i* = 0, 1, 2, 3 representing heifers and parities 1, 2 and 3 of cows in models (1) and (2); *i* = 1, 2 representing fertility traits 1 and 2 in models (4) and (5)); ***β***_***i***_ was the vector of fixed effects for the *i*th trait, including age group (in terms of age at first insemination, divided into 270–439 days, 440–469 days, 470–499 days, 500–529 days and 530–900 days), herd-year of calving (for ICF and DO) or herd-year of first insemination within parity (for IFL, CR, AIS and NRR56), year-month of calving (for ICF and DO) or year-month of first insemination within parity (for IFL, CR, AIS and NRR56), AI technician, sexed semen (coded as 1 for the use of sexed semen and 0 for the use of non-sexed semen), and parity (available only for models (3) and (5); ***a***_***i***_ was the vector of random additive genetic effects for the *i*th trait; ***pe***_***i***_ was the vector of permanent environmental effects for the *i*th trait (available only for models (3) and (5)); and ***e***_***i***_ was the vector of random residual effects for the *i*th trait. ***X***_***i***_, ***Z***_***i***_ and ***W***_***i***_ were the incidence matrices connecting ***β***_***i***_ **,** ***a***_***i***_ and ***pe***_***i***_ **to** ***y***_***i***_. It was assumed that $$ \left[\begin{array}{c}\hfill {\boldsymbol{a}}_{\mathbf{1}}\hfill \\ {}\hfill \vdots \hfill \\ {}\hfill {\boldsymbol{a}}_{\boldsymbol{n}}\hfill \end{array}\right]\sim N\left(\mathbf{0},\boldsymbol{A}\otimes \left[\begin{array}{ccc}\hfill {\sigma}_{a_1}^2\hfill & \hfill \cdots \hfill & \hfill {\sigma}_{a_1{a}_n}\hfill \\ {}\hfill \hfill & \hfill \ddots \hfill & \hfill \vdots \hfill \\ {}\hfill \hfill & \hfill \hfill & \hfill {\sigma}_{a_n}^2\hfill \end{array}\right]\right) $$, $$ \left[\begin{array}{c}\hfill {\boldsymbol{pe}}_1\hfill \\ {}\hfill \vdots \hfill \\ {}\hfill {\boldsymbol{pe}}_{\boldsymbol{n}}\hfill \end{array}\right]\sim N\left(\mathbf{0},\boldsymbol{I}\otimes \left[\begin{array}{ccc}\hfill {\sigma}_{pe_1}^2\hfill & \hfill \cdots \hfill & \hfill {\sigma}_{pe_1{pe}_n}\hfill \\ {}\hfill \hfill & \hfill \ddots \hfill & \hfill \vdots \hfill \\ {}\hfill \hfill & \hfill \hfill & \hfill {\sigma}_{pe_n}^2\hfill \end{array}\right]\right) $$, $$ \left[\begin{array}{c}\hfill {\boldsymbol{e}}_{\mathbf{1}}\hfill \\ {}\hfill \vdots \hfill \\ {}\hfill {\boldsymbol{e}}_{\boldsymbol{n}}\hfill \end{array}\right]\sim N\left(\mathbf{0},\boldsymbol{I}\otimes \left[\begin{array}{ccc}\hfill {\sigma}_{e_1}^2\hfill & \hfill \cdots \hfill & \hfill {\sigma}_{e_1{e}_n}\hfill \\ {}\hfill \hfill & \hfill \ddots \hfill & \hfill \vdots \hfill \\ {}\hfill \hfill & \hfill \hfill & \hfill {\sigma}_{e_n}^2\hfill \end{array}\right]\right) $$.

In the above distributions, ***A*** is the matrix of additive genetic relationships between individuals in the pedigree; ***I*** is the identity matrix; ${\sigma}_{a_i}^2$, ${\sigma}_{pe_i}^2$ and ${\sigma}_{e_i}^2$ were the additive genetic variance, permanent environmental variance and residual variance of the *i*th trait, respectively; ${\sigma}_{a_i{a}_j}$, ${\sigma}_{pe_i p{e}_j}$ and ${\sigma}_{e_i{e}_j}$ (*i* ≠ *j*) were the additive genetic covariance, permanent environmental covariance and residual covariance between the *i*th trait and the *j*th trait, respectively.

The (co)variance components in the above models were estimated using average information-restricted maximum likelihood (AI-REML) implemented in the DMU package [20]. The asymptotic standard errors of the variances and covariances were obtained from the average information matrix. Standard errors of heritabilities and correlations were calculated according to an expansion of the Taylor series, as previously described [21].

## Results

The descriptive statistics for female fertility traits regarding heifers and cows are presented in Table 1. In general, all fertility capacities of the heifers were superior to those of the cow: IFL were 47.4 days shorter, CR was 27.0% higher, NRR56 was 14.8% higher, and AIS was 1.035 less. For all traits of both heifers and cows, large phenotypic variations were observed.

**Table 1:** Descriptive statistics of female fertility traits in a Chinese Holstein population

Trait^a^	N	Mean	SD	Minimum	Maximum
IFLh	88,647	32.1	69.2	0	365
CRh	88,647	0.626	0.484	0	1
AISh	88,647	1.742	1.286	1	8
NRR56h	88,647	0.732	0.443	0	1
ICF	126,985	82.1	37.3	20	230
DO	126,985	161.6	104.9	20	595
IFLc	126,985	79.5	100.1	0	365
CRc	126,985	0.356	0.479	0	1
AISc	126,985	2.777	1.994	1	8
NRR56c	126,985	0.584	0.493	0	1

^a^ *IFL* interval from first to last insemination, *CR* conception rate at first insemination, *AIS* number of inseminations per conception, *NRR56* non-return rate at 56 days after first insemination, *ICF* interval from calving to first insemination, *DO* days open. For traits expressed in both heifers and cows, a suffixes h (for heifers) or c (for cows) was attached to the trait abbreviations

Estimates of the variance components and heritabilities of fertility traits using a multi-trait model and a single-trait model are shown in Table 2. Estimates of heritability of female fertility traits were generally low, ranging from 0.005 (first parity of cows for NRR56) to 0.102 (first parity of cows for ICF). The approximate standard errors for the heritability estimates ranged from 0.001 to 0.011. The estimated heritabilities all differed significantly from zero. As shown in Table 2, binary traits (CR and NRR56) did not exhibit large differences in terms of variance components between heifers and cows. However, for IFL and AIS, the additive variances of heifers (56.6 for IFLh and 0.018 for AISh) were only approximately one = fifth those of cows (257.8 for IFLc and 0.095 for AISc in an analysis of data pooled over parities). For cow traits, additive variances for ICF, DO and IFLc in the first parity were higher than those in the second and third parities, but CRc, AISc and NRR56c exhibited no clear trends. In addition, the three interval traits of cows (ICF, IFL and DO) had higher heritabilities (ranging from 0.027 to 0.083) than the other three traits (ranging from 0.006 to 0.026). On the other hand, the heritabilities of the four heifer traits did not differ dramatically (ranging from 0.008 to 0.013).

**Table 2:** Variance components^a^ using a multi-trait model and a single-trait model

Parameter^b^	IFL	CR	AIS	NRR56	ICF	DO
${\sigma}_{a_0}^2$	56.6	1.851 E-03	0.018	1.352 E-03		
${\sigma}_{a_1}^2$	325.8	2.921 E-03	0.101	1.120 E-03	120.5	539.7
${\sigma}_{a_2}^2$	282.3	1.937 E-03	0.100	1.292 E-03	69.1	380.0
${\sigma}_{a_3}^2$	238.0	2.821 E-03	0.101	2.697 E-03	59.5	396.6
${\sigma}_{e_0}^2$	4326.4	0.178	1.392	0.162		
${\sigma}_{e_1}^2$	9398.3	0.204	3.390	0.210	1059.5	10,520.4
${\sigma}_{e_2}^2$	9073.3	0.187	3.642	0.217	818.8	9862.0
${\sigma}_{e_3}^2$	8944.9	0.189	3.642	0.221	798.6	9660.6
${\widehat{h}}_0^2$	0.013(0.003)	0.010(0.002)	0.013(0.002)	0.008(0.002)		
${\widehat{h}}_1^2$	0.034(0.005)	0.014(0.003)	0.029(0.005)	0.005(0.002)	0.102(0.008)	0.049(0.006)
${\widehat{h}}_2^2$	0.030(0.005)	0.010(0.003)	0.027(0.005)	0.006(0.003)	0.078(0.009)	0.037(0.006)
${\widehat{h}}_3^2$	0.026(0.006)	0.015(0.005)	0.027(0.006)	0.012(0.005)	0.069(0.011)	0.039(0.007)
${\sigma}_{a_{123}}^2$	257.8	2.314 E-03	0.095	1.215 E-03	86.5	433.8
${\sigma}_{pe_{123}}^2$	804.5	5.285 E-03	0.317	5.664 E-03	41.3	1173.8
${\sigma}_{e_{123}}^2$	8438.8	0.191	3.214	0.210	911.3	9034.1
${\widehat{h}}^2$_123_	0.027(0.004)	0.012(0.002)	0.026(0.003)	0.006(0.001)	0.083(0.006)	0.041(0.005)

^a^ ${\sigma}_{a_i}^2\left( i=0,1,2,3\right)$ = additive genetic variance of *i*th parity using multi-trait models (model 1); ${\sigma}_{e_i}^2\left( i=0,1,2,3\right)$ = residual variance of *i*th parity using multi-trait models (model 1); ${\widehat{h}}_i^2$ = estimated heritability of *i*th parity using multi-trait models (model 1); ${\sigma}_{a_{123}}^2$ = additive genetic variance of a cow trait over parities using single-trait models (model 4); ${\sigma}_{pe123}^2$ = permanent environmental variance of a cow trait over parities using single-trait models; ${\sigma}_{e_{123}}^2$ = residual variance of a cow trait over parities using single-trait models (model 4); ${\widehat{h}}_{123}^2$ = estimated heritability of acow trait over parities using single-trait models

^b^ *IFL* interval from first to last insemination, *CR* conception rate at first insemination, *AIS* number of inseminations per conception, *NRR56* non-return rate at 56 days after first insemination, *ICF* interval from calving to first insemination, *DO* days open

The genetic and residual correlations between parities of the performances of a trait using the multi-trait model are presented in Table 3. Generally, the genetic correlations between the performances in heifers and in cows were moderate but far from one, ranging from 0.25 for AIS between heifers and cows at the third parity to 0.74 for NRR56 between heifers and cows at the second parity. However, the genetic correlations between parities of cows were generally high, except for those between the third parity and the other two parities which were 0.63 and 0.66 and had large standard errors, the other genetic correlation ranged from 0.87 to 1. On the other hand, the residual correlations between heifers and cows and between parities of cows were very low, ranging from −0.002 to 0.14, indicating small permanent effect on fertility traits. Since all fertility traits had low heritabilities, the phenotypic correlations (not presented) were just very slightly higher than the residual correlations.

**Table 3:** Correlations^a^ between performances of a trait in different parities using a multi-trait model

Trait^b^	IFL	CR	AIS	NRR56	ICF	DO
${r}_{a_0,{a}_1}$	0.64 (0.09)	0.45(0.15)	0.48(0.11)	0.65(0.18)		
${r}_{a_0,{a}_2}$	0.49 (0.12)	0.52(0.18)	0.40(0.13)	0.74(0.22)		
${r}_{a_0,{a}_3}$	0.61 (0.14)	0.25(0.22)	0.55(0.14)	0.37(0.23)		
${r}_{a_1,{a}_2}$	0.89 (0.06)	1.00(0.11)	0.92(0.06)	0.99(0.24)	0.91(0.03)	0.89(0.05)
${r}_{a_1,{a}_3}$	0.93 (0.08)	0.66(0.19)	0.92(0.08)	0.92(0.23)	0.92(0.04)	0.96(0.05)
${r}_{a_2,{a}_3}$	0.94 (0.07)	0.63(0.21)	0.94(0.07)	0.87(0.24)	0.96(0.04)	0.96(0.05)
${r}_{e_0,{e}_1}$	0.06 (0.01)	0.03(0.004)	0.07(0.005)	0.004(0.004)		
${r}_{e_0,{e}_2}$	0.04 (0.01)	0.01(0.01)	0.05(0.01)	−0.002(0.005)		
${r}_{e_0,{e}_3}$	0.04 (0.01)	0.01(0.01)	0.05(0.01)	0.01(0.01)		
${r}_{e_1,{e}_2}$	0.09 (0.01)	0.02(0.01)	0.09(0.01)	0.02(0.01)	0.05(0.01)	0.12(0.01)
${r}_{e_1,{e}_3}$	0.07 (0.01)	0.02(0.01)	0.08(0.01)	0.02(0.01)	0.01(0.01)	0.08(0.01)
${r}_{e_2,{e}_3}$	0.11 (0.01)	0.04(0.01)	0.13(0.01)	0.03(0.01)	0.07(0.01)	0.14(0.01)

^a^ ${r}_{a_i,{a}_j}$ = genetic correlation between performances of a trait in parities *i* and *j*, ${r}_{e_i,{e}_j}$ = residual correlation between performances of a trait in parities *i* and *j*. Phenotypic correlations were similar to residual correlations (not presented)

^b^ *IFL* interval from first to last insemination, *CR* conception rate at first insemination, *AIS* number of inseminations per conception, *NRR56* non-return rate at 56 days after first insemination, *ICF* interval from calving to first insemination, *DO* days open

Table 4 presents the genetic and phenotypic correlations between heifer traits. The genetic correlations between IFLh, CRh, AISh and NRR56h were strong. The absolute values of these genetic correlations ranged from 0.63 (negative between IFLh and NRR56h) to 0.94 (negative between CRh and AISh). The phenotypic correlations between these traits were moderate to high. The absolute values of these phenotypic correlations ranged from 0.26 (negative between IFLh and NRR56h) to 0.80 (negative between IFLh and AISh).

**Table 4:** Genetic (above the diagonal) and phenotypic (below the diagonal) correlations between heifer traits using a two-trait model

Trait^a^	IFLh	CRh	AISh	NRR56h
IFLh		−0.86	0.85	−0.63
		(0.06)	(0.04)	(0.12)
CRh	−0.58		−0.94	0.84
	(2.24 E-03)		(0.03)	(0.06)
AISh	0.80	−0.70		−0.87
	(1.24 E-03)	(1.72 E-03)		(0.06)
NRR56h	−0.26	0.76	−0.56	
	(3.14 E-03)	(1.42 E-03)	(2.32 E-03)	

^a^ *IFLh* interval from first to last insemination in heifers, *CRh* conception rate at first insemination in heifers, *AISh* number of inseminations per conception in heifers, *NRR56h* non-return rate within 56 days after first insemination in heifers

The genetic and phenotypic correlations between cow traits, estimated using data pooled over three parities, are shown in Table 5. There were strong correlations between DO, IFLc, CRc and AISc. The absolute values of genetic correlations between the four traits ranged from 0.82 (positive between DO and AISc) to 0.98 (negative between DOc and IFLc), and the phenotypic correlations ranged from 0.55 (negative between DO and CRc) to 0.97 (positive between DOc and IFLc). ICF had high genetic correlation and moderate phenotypic correlation with DO, while NRR56c had moderate genetic and phenotypic correlations with CRc and AISc.

**Table 5:** Genetic (above the diagonal) and phenotypic (below the diagonal) correlations between cow traits using a two-trait model

Trait^a^	ICF	DO	IFLc	CRc	AISc	NRR56c
ICF		0.77	0.48	−0.43	0.35	0.40
		(0.03)	(0.07)	(0.09)	(0.07)	(0.11)
DO	0.33		0.98	−0.87	0.82	−0.01
	(2.67 E-03)		(1.57 E-03)	(0.04)	(0.03)	(0.13)
IFLc	2.06 E-03	0.97		−0.90	0.90	−0.15
	(3.00 E-03)	(2.33E-04)		(0.04)	(0.02)	(0.14)
CRc	0.01	−0.55	−0.58		−0.95	0.31
	(2.93 E-03)	(2.00 E-03)	(1.88 E-03)		(0.03)	(0.14)
AISc	1.61 E-03	0.81	0.85	−0.63		−0.51
	(3.00 E-03)	(1.01 E-03)	(7.92 E-04)	(1.70 E-03)		(0.11)
NRR56c	0.03	−0.18	−0.21	0.59	−0.42	
	(2.91 E-03)	(2.76 E-03)	(2.71 E-03)	(1.83 E-03)	(2.35 E-03)	

^a^ *ICF* interval from calving to first insemination, *DO* days open, *IFLc* interval from first to last insemination in cows, *CRc* conception rate at first insemination in cows, *AISc* number of inseminations per conception in cows, *NRR56c* non-return rate at 56 days after first insemination in cows

## Discussion

This study investigated the relationships among parities and variance components in each parity for female fertility traits in Chinese Holsteins. In addition, the present study estimated genetic and phenotypic correlations between heifer traits and between cow traits. The results obtained from the present study will be useful for genetic evaluations in dairy cattle breeding programs, particularly for developing a routine genetic evaluation system for female fertility traits in Chinese Holstein population.

The average performances of fertility traits in the present study were similar to those of other populations in general [6, 12, 22]. It was also observed that all the fertility performances of heifers were better than those of cows, consistent with previous studies [23]. The differences in the reproductive physiology between maiden heifers and cows could be caused by first calving and the negative energy balance resulting from high milk yield [13]. In addition, the larger amount of censored data for cows (13.92%) than for heifers (5.92%) could imply that cows had poorer fertility conditions than heifers.

The heritabilities obtained in the present study were consistent with the previous estimates for fertility traits in various populations [6, 12, 24]. For example, Veerkamp and Beerda [25] reviewed the heritabilities estimated in 17 studies and reported that the average heritability estimates for IFL, CR, AIS, NRR56, ICF and DO were less than 5%. Similarly, in the present study, the estimates of heritabilities of IFL, CR, AIS and NRR56 in various parities ranged from 0.005 to 0.034. Furthermore, the estimates of heritabilities of ICF (0.069-0.102) and DO (0.037-0.049) were higher than those of the other traits, which was consistent with previous studies [12, 24].

For traits defined in both heifers and cows, variance components for IFL and AIS differed considerably between heifers and cows, and heritability estimates of the two heifer traits were much lower than those of the corresponding cow traits. These results were consistent with previous observations in Canadian Holsteins [6], Brown Swiss [23] and US Holsteins [26]. Moreover, genetic correlations between the performances of heifers and cows were moderate but far from one, consistent with previous studies [6, 14, 23, 26]. These results further indicate that traits in heifers and cows should be treated as different but genetically correlated traits in genetic evaluations. In a breeding program, both heifer and cow traits should be considered. In addition, information for heifer traits is useful for early selection, as heifer traits can be measured during a relatively early period of life.

Genetic correlations between the performances of a trait across parities of cows were high (over 0.87) with the exception for CRc, for which the genetic correlation between the first and second parities was 0.66 and between the second and third parities was 0.63 but with a relatively large standard error. The results were consistent with previous literature [15, 23, 27]. Despite the high genetic correlation, the additive genetic variances of ICFc and DOc in the first parity were substantially larger than those in later parities. The additive genetic variance of IFLc in the first parity was also clearly larger than those in later parities. The differences in additive genetic variances led to differences in heritabilities between the first parity and the other two parities, but the differences in heritabilities were smaller than the differences in additive genetic variances. Differences in additive genetic variances and heritabilities between parities of cows for fertility traits have also been reported in previous studies [15, 23]. The results from the present study suggest that even if there is a high genetic correlation between parities of cows, the performance of a trait in the first parity should be treated different from those in later parities in genetic evaluations, especially for ICF, DO and IFL. To increase reliability and reduce bias in routine genetic evaluations for female fertility traits, Nordic countries have treated parities from 1 to 3 as separate traits instead of repeated observations since May 2015 (http://www.nordicebv.info).

Although the genetic correlations among traits varied substantially, moderate or strong correlations were obtained between traits in groups with similar definitions of fertility functions. Therefore, four groups of traits defined based on reproductive biology highly consistent with the patterns of genetic correlations. The first group of traits measured the abilities of the maiden heifers to conceive and maintain pregnancy, including IFLh, CRh, AISh and NRR56h, which had genetic correlations ranging from 0.63 to 0.94 in absolute values. The second trait group measured the abilities of lactating cows to recycle after calving, indicated by ICF in the present study. The third group of traits described the capacities of cows to conceive and maintain pregnancy, including IFLc, CRc, AISc and NRR56c. Most genetic correlations between these traits were moderate to high, ranging from 0.51 to 0.95 in absolute values, except for the correlations of NRR56 with IFLc (−0.15) and CRc (0.31). The fourth group of traits was DO, which measured the combined abilities to recycle, conceive and maintain pregnancy. This grouping of traits is consistent with the current Interbull concepts of grouping fertility traits.

The traits IFL, CR, AIS and NRR56 all reflect the ability to conceive and maintain pregnancy. According to the estimated genetic parameters, the expected responses to selection given the same selection intensity can be calculated. Among these traits, selection for IFL would achieve the largest genetic progress in the ability to conceive and maintain pregnancy in both heifers and cows. Since IFL had a very high genetic correlation with CR and AIS, it is not necessary to include CR and AIS in a fertility index that contains IFL. NRR56, which measures the capacity for conception and early embryo loss, had a low genetic correlation with IFL for cows. It would be reasonable to include NRR56 in the fertility index. There is no need to include DO in the fertility index given its component traits, ICF and IFL, are already in the index. Thus, IFL, NRR56 and ICF are recommended to be included in the selection index of the Chinese Holstein population.

The present study also estimated genetic and phenotypic correlations between a pair of traits in each parity, using a two-trait model (results not shown). Compared with the results from the analysis using pooled data over parities and treating them as repeated records, the patterns of estimated correlations in each parity were somewhat different from those estimated using the repeatability model (model 5). Moreover, the estimated correlations were not stable across different parities. One possible reason could be bias caused by selection on previous parities, especially the selection for heifer fertility. To avoid this bias, a multi-trait model, which used data from all parities and treated fertility traits from different parities as different traits, was implemented to estimate correlations between a pair of traits for each parity. However, this multi-trait model had difficulty reaching convergence. Therefore, only correlations estimated using data pooled over parities and treating them as repeated records were presented in this study.

For traits with binary phenotypes, a linear model was used in the present study due to low computational requirements. The results showed that the heritability estimates of binary traits were generally lower than those of interval traits measuring conception abilities, consistent with previous studies [6, 8]. Many sophisticated models, such as the threshold model and the generalized linear mixed model, have been proposed for the genetic evaluation of categorical fertility traits. Differences between linear and non-linear models were negligible when phenotypes were polychotomous or the incidences of the binary response were not extreme, e.g. between 25 and 75% [28]. Sun and Su [7] reported that probit and logit models performed slightly better than linear models in terms of the prediction of breeding values for non-return rate and success in first insemination, but the correlations between estimated breeding values from different models were high (up to 0.99). Furthermore, a study assessing NRR56 and ICF using a bivariate threshold-linear and a bivariate-linear model showed that no difference was observed between two models in predictive ability [29]. Nevertheless, due to its intensive computational costs and possible numerical difficulties, the threshold model was not recommended for practical use [29]. Moreover, a simulation study comparing computing aspects of linear and non-linear models illustrated that the computing cost of a threshold model analysis was three to five times larger than that of a linear model [7]. Hence, linear models are widely used in practical routine genetic evaluations of categorical fertility traits in many countries, which is also the reason that a linear model was used in the present study. In fact, four types of generalized linear mixed models, including a probit animal model, a probit sire model, a logit animal model and a logit sire model, were also applied for CR and NRR56 (results not shown). The estimates of heritability from these models were similar with those of the linear animal models. Therefore, only the results of the linear animal models were presented here.

One large challenge in genetic evaluations of fertility interval traits is the presence of censoring, which reflects data truncated through culling or fertility events still in progress. Regarding censored data as uncensored or simply excluding them from the dataset would favor bulls with many daughters having censored records [30], and would fail to account for selection bias [14]. A simple strategy to reduce bias is to add a penalty to censored records. In previous studies of Danish Holsteins, a penalty of 21 days was added to censored ICF, IFL and DO, and a penalty of 1 was added to censored AIS, assuming that cows failing to become pregnant would conceive when provided an extra estrus cycle [19, 24, 31]. In the present study, a penalty of the expected value for a fertility trait was added to a censored record, assuming that the success of an insemination was independent of the result of previous inseminations. The methods for adding a penalty to censored records are simple and offer a potentially sub-optimal strategy to manage censored data. Other methods, such as proportional hazard models [24, 32], may be more efficient for handling data that include censored records. However, such models also have some limitations, e.g., high computational demand or extra work to obtain animal model solutions. Moreover, choosing the optimum editing criteria using data truncation with different threshold limits could also be an alternative strategy for dealing with censored data [30].

## Conclusions

The parameters estimated in the present study will facilitate the development of a genetic evaluation system for fertility traits to improve the female fertility abilities of Chinese Holsteins. The performances of a trait in heifers and cows were significantly different in genetic and should be considered as distinct but correlated traits in genetic evaluations. In addition, even though high genetic correlations between the performances of a trait in different parities of cows, additive genetic variances and heritabilities differed between the first parity and the later parities, which suggests that the performances of a trait in the first and the later parities should be treated as different traits in genetic evaluations. Generally, the estimated heritabilities of all female fertility traits were low, but selection to improve fertility is promising since the genetic variations are large. IFL, NRR56 and ICF are recommended to be included in the selection index of the Chinese Holstein population.

## Abbreviations

AI-REML: Average information-restricted maximum likelihood
AIS: Number of inseminations per conception
CR: Conception rate at first insemination
DO: Days open
ICF: Interval from calving to first insemination
IFL: Interval from first to last insemination
MACE: Multiple across-country evaluation
NRR56: Non-return rate within 56 days after first insemination
